# Locus‐specific concordance of genomic alterations between tissue and plasma circulating tumor DNA in metastatic melanoma

**DOI:** 10.1002/1878-0261.12391

**Published:** 2018-12-07

**Authors:** Leslie Calapre, Tindaro Giardina, Cleo Robinson, Anna L. Reid, Zeyad Al‐Ogaili, Michelle R. Pereira, Ashleigh C. McEvoy, Lydia Warburton, Nicholas K. Hayward, Muhammad A. Khattak, Tarek M. Meniawy, Michael Millward, Benhur Amanuel, Melanie Ziman, Elin S. Gray

**Affiliations:** ^1^ School of Medical and Health Sciences Edith Cowan University Joondalup Australia; ^2^ Anatomical Pathology PathWest Laboratory Medicine QEII Medical Centre Nedlands Australia; ^3^ School of Biomedical Science University of Western Australia Crawley Australia; ^4^ Department of Molecular Imaging and Therapy Service Fiona Stanley Hospital Murdoch Australia; ^5^ Department of Medical Oncology Sir Charles Gairdner Hospital Nedlands Australia; ^6^ QIMR Berghofer Medical Research Institute Brisbane Australia; ^7^ School of Medicine University of Western Australia Crawley Australia; ^8^ Department of Medical Oncology Fiona Stanley Hospital Murdoch Australia

**Keywords:** circulating tumor DNA, concordance, melanoma, promoter

## Abstract

Circulating tumor DNA (ctDNA) may serve as a surrogate to tissue biopsy for noninvasive identification of mutations across multiple genetic loci and for disease monitoring in melanoma. In this study, we compared the mutation profiles of tumor biopsies and plasma ctDNA from metastatic melanoma patients using custom sequencing panels targeting 30 melanoma‐associated genes. Somatic mutations were identified in 20 of 24 melanoma biopsies, and 16 of 20 (70%) matched‐patient plasmas had detectable ctDNA. In a subgroup of seven patients for whom matching tumor tissue and plasma were sequenced, 80% of the mutations found in tumor tissue were also detected in ctDNA. However, *TERT* promoter mutations were only detected by ddPCR, and promoter mutations were consistently found at lower concentrations than other driver mutations in longitudinal samples. *In vitro* experiments revealed that mutations in promoter regions of *TERT* and *DPH3* are underrepresented in ctDNA. While the results underscore the utility of using ctDNA as an alternative to tissue biopsy for genetic profiling and surveillance of the disease, our study highlights the underrepresentation of promoter mutations in ctDNA and its potential impact on quantitative liquid biopsy applications.

**Abbreviations**
cfDNA: cell‐free DNA
ctDNA: circulating tumor DNA
ddPCR: digital droplet PCR
FDG‐PET/CT: ^18^F‐labeled fluorodeoxyglucose positron emission tomography/computed tomography
FFPE: formalin‐fixed paraffin embedded tissue
MTB: metabolic tumor burden
NGS: next‐generation sequencing

## 1. Introduction

The use of targeted therapeutic agents and immune checkpoint inhibitors has improved the survival of metastatic melanoma patients in recent years (Luke *et al*., 2017). Current treatment strategies employ various systemic agents, often used in succession, that are dependent on the genetic landscape of the tumor (Ascierto *et al*., 2013; Chen *et al*., 2016; Larkin *et al*., 2014; Luke *et al*., 2017; Ribas *et al*., 2015; Santiago‐Walker *et al*., 2016). Treating physicians are confronted with new challenges, such as stratifying patients for appropriate treatments and monitoring long‐term responders for progression. Consequently, reliable methods for monitoring disease progression and treatment response or resistance are necessary.

Circulating tumor DNA (ctDNA), which is shed into the blood as a result of tumor cell apoptosis and necrosis, has been shown to have potential clinical utility for molecular classification (Haselmann *et al*., 2018), prognostication (Ascierto *et al*., 2013; Gray *et al*., 2015; Knol *et al*., 2016; Sanmamed *et al*., 2015), and monitoring patient response to therapy (Girotti *et al*., 2016; Gray *et al*., 2015; Lee *et al*., 2017; Schreuer *et al*., 2016; Wong *et al*., 2017) in melanoma. Plasma ctDNA has also been shown to capture clonal evolution, via identification of mutations that mediate resistance to BRAF inhibitors (Girotti *et al*., 2016; Gray *et al*., 2015). Moreover, the analysis of plasma and multiple metastatic deposits in two melanoma patients indicated that ctDNA can reflect the genetic heterogeneity of various subclones across multiple tumors (Wong *et al*., 2017). Thus, ctDNA appears to be a useful biomarker for patient surveillance during treatment, acting as a potential surrogate to tissue biopsy and providing a comprehensive snapshot of the molecular diversity of metastases. Nevertheless, the detection rate of ctDNA in melanoma patients and concordance of mutations between plasma and tissue still requires further study, especially beyond detection of *BRAF* mutations (Calapre *et al*., 2017).

Based on their somatic mutation profiles, melanomas can be divided into four genomic subtypes: *BRAF*,* RAS* (N/H/K), *NF1*, and triple wild‐type (WT) (TCGA, 2015). Recurring hotspot mutations in the V600 codon of *BRAF* or Q61 codon of *NRAS* are the most prevalent and occur in approximately 35–50% and 10–25% of melanomas, respectively (Pollock *et al*., 2002; TCGA, 2015; Tsao *et al*., 2012). Most ctDNA studies thus far have only analyzed *BRAF* mutant cases (Ascierto *et al*., 2013; Girotti *et al*., 2016; Gray *et al*., 2015; Knol *et al*., 2016; Schreuer *et al*., 2016). These studies have remarked on the high fidelity of *BRAF* mutant ctDNA to reflect disease burden and tumor status of patients prior to and during treatment (Ascierto *et al*., 2013; Girotti *et al*., 2016; Gray *et al*., 2015; Knol *et al*., 2016; Schreuer *et al*., 2016). However, it is imperative to ascertain the detection rate and kinetics of other common melanoma‐associated mutations to determine whether they can be effectively used for patient surveillance, particularly in *BRAF* WT cases.

In this study, we identified tumor mutations using a custom next‐generation sequencing (NGS) panel targeting melanoma‐specific genes in a cohort of metastatic melanoma patients and determined the detection rate of ctDNA by targeting mutations identified in each patient's tumor. We performed sequencing of a set of paired melanoma tissue biopsies and circulating free DNA (cfDNA) to determine the level of concordance of mutations across these two compartments. Furthermore, we evaluated the suitability of various mutated loci for monitoring ctDNA in patients undergoing systemic therapies. Finally, we performed *in vitro* experiments to evaluate whether mutations in promoter regions are underrepresented in cfDNA.

## 2. Materials and methods

### 2.1. Patients

Metastatic melanoma patients were enrolled in the study between 2013 and 2016 at Sir Charles Gairdner Hospital (SCGH) and Fiona Stanley Hospital (FSH) in Perth, Western Australia. Written informed consent was obtained from all patients under approved Human Research Ethics Committee protocols from Edith Cowan University (No. 11543) and Sir Charles Gairdner Hospital (No. 2007‐123). The study methodologies conformed to the standards set by the Declaration of Helsinki.

### 2.2. Tissue analysis

Tissue biopsies were retrospectively tested for mutation profile. The tissue biopsies were included if obtained prior to therapy initiation, with no systemic treatment during that period. Hematoxylin and eosin (H&E)‐stained sections were assessed by a pathologist and the percentage of tumor cells estimated. Microdissection was performed when the neoplastic cell content was below 50%. DNA was isolated using QIAamp Tissue FFPE Kits (Qiagen, Hilden, Germany) as per the manufacturer's instructions. FFPE gDNA was stored at 4 °C until processed for targeted NGS (Supporting Information).

### 2.3. Plasma sample preparation and cfDNA extractions

Blood samples were collected prior to initiation of treatment into EDTA vacutainer or Cell‐Free DNA BCT^®^ (Streck, La Vista, NE, USA) tubes and stored at 4 °C. Plasma was separated within 24 h by centrifugation at 300 g for 20 min, followed by a second centrifugation at 4700 g for 10 min and then stored at −80 °C until extraction. cfDNA was isolated from 1 to 5 mL of plasma using QIAamp Circulating Nucleic Acid Kits (Qiagen, Hilden, Germany) as per the manufacturer's instructions. cfDNA was eluted in 40 μl AVE buffer (Qiagen, Hilden, Germany) and stored at −80 °C until ctDNA quantification by droplet digital PCR (ddPCR) (Supporting Information) or processed for sequencing using a QIASeq Targeted DNA Custom Panel (CDHS‐12967Z‐1243) (Supporting Information).

### 2.4. Metabolic tumor burden analysis

^18^F‐labeled fluorodeoxyglucose positron emission tomography/computed tomography (FDG‐PET/CT) scans were performed on combined PET/CT scanners at approved nuclear radiology centers in Perth, Western Australia. After a minimum fasting period of 6 h, patients were injected with 5 MBq pr. kg ±10% of ^18^FDG (minimum 200 MBq and maximum 600 MBq). PET was performed on patients with serum glucose levels below 11 nmol·L^−1^ at an acquisition time of 3 min per bed position. To determine anatomical location and for attenuation correction purposes, a whole‐body low‐dose computed tomography scan was performed. All images were reviewed retrospectively and independently by an experienced nuclear medicine physician, blinded to the ctDNA analysis. Analysis was conducted on a Siemens Syngo via workstation (Siemens Healthcare GMbH, Erlangen, Germany) reporting the global total lesion glycolysis (TLG), which combines volumetric and metabolic information (Chen *et al*., 2012; Kim *et al*., 2013) and can provide a better evaluation of Metabolic tumor burden (MTB).

### 2.5. Targeted amplicon sequencing and bioinformatics of tumor tissue

Tissue biopsy mutation profiles were identified by targeted NGS using a customized panel of 30 melanoma‐associated genes (Illumina, San Diego, CA, USA) with 950 amplicons and an Illumina MiSeq instrument. Forward and reverse strand NGS libraries were prepared using the customized melanoma panel according to the manufacturer's instructions. In brief, forward and reverse oligonucleotide pools were hybridized to DNA samples overnight. Hybridized samples were then ligated, extended, and amplified with unique index sequences (barcodes) and sequencing adaptors. Amplified libraries were purified using Agencourt AMPure XP magnetic beads (Beckman Coulter, Brea, CA, USA), first at a ratio of 1 (library):1 (beads), as per the manufacturer's protocol, followed by a second round of purification at a library to bead ratio of 1.25 : 1. Library DNA concentrations were quantified using a Qubit 3.0 fluorometer. Libraries were normalized to 4 nmol·L^−1^ in EBT buffer, pooled, and sequenced on a MiSeq instrument (Illumina). Sequence alignment and variant calling were performed by illumina miseq reporter software (version 2.4, Illumina). Genomic variants were annotated using the illumina variant studio 2.2 software (Illumina). Variants with allele frequency (VAF) >3% and that passed the software quality parameters were considered true mutations. Polymorphisms and synonymous mutations were excluded, and the minimum read depth was set at 500. The polyPhen score, which represent the probability of the impact of an amino acid substitution on the protein structure and function, was indicated for each variant.

### 2.6. Sequencing and bioinformatics of cfDNA

QIASeq Targeted DNA Custom Panel (CDHS‐12967Z‐1243) containing the same panel of 30 melanoma‐associated genes with similar regions of interest was used to determine the mutational profile of a subset of ctDNA samples. Isolated DNA was quantified with Quant‐iT dsDNA High‐Sensitivity Assay Kits (Life Technologies, Carlsbad, CA, USA) or Quant‐iT dsDNA BR Assay Kits (Life Technologies).

QIASeq Targeted DNA Panel Kits were used for library generation and target enrichment. Fragment size distribution of the libraries was determined with an Agilent Bioanalyzer using a DNA 7500 chip (Agilent Technologies, Santa Clara, CA, USA). Library quantification was performed using a KAPA Library Quantification Kit for Illumina Platforms (Roche, Basel, Switzerland). Indexed sample libraries were equimolarly pooled and sequenced on an Illumina NextSeq sequencer using a NextSeq 500 Mid Output v2 Kit (300 cycles). Data analysis including alignment to the reference genome and variant calling was carried out using QIAseq Target DNA online portal and ingenuity variant analysis software (Qiagen, Hilden, Germany).

Concordance was defined as detecting an identical single nucleotide variation (SNV) in plasma relative to tissue in regions that were covered by both targeted amplicon sequencing panels used for NGS of tissue and plasma biopsies. Discordance was defined as SNVs detected only in plasma or tumor tissue. Concordance of SNVs found in plasma that were detectable in tissue by NGS (Fig. S2) was calculated for each tissue/plasma pair using the formula:

Concordance(%)=(x÷y)×100

*x *= number of variants confirmed in plasma and tissue; *y *= total number of variants detected in plasma.

### 2.7. Droplet digital PCR

Commercially available and/or customized probes were used to analyze ctDNA by ddPCR. Droplets were generated using an Automatic Droplet generator QX200 AutoDG (Bio‐Rad, Hercules, CA). Amplifications were performed using cycling conditions previously described (Gray *et al*., 2015; McEvoy *et al*., 2017). For *DPH3* mutation analysis, the following probe and primer design was used: forward primer sequence: GGG CTC GGC ATC ATC AG, reverse primer sequence: CCG CTA CCG GTT ATC CAT TT, DPH3 c.C8T probe: /56‐FAM/TAG CTC TTC/ZEN/CGG CGC A/3IABkFQ/, DPH3 WT probe: /5HEX/TAG CCC TTC/ZEN/CGG CGC A/3IABkFQ/, from Integrated DNA Technologies (Coralville, IO, USA). Primers and probes for TERT ctDNA analysis were as previously reported (McEvoy *et al*., 2017). Levels of ctDNA per loci were defined based on the level of false‐positive droplets in at least 12 healthy controls (Table S5).

### 2.8. Cell culture

UACC62 cells were obtained from NCI's Development Therapeutics Program; 1205Lu cells were obtained from Meehard Herlyn, The Wistar Institute; and C037 and A07 cells were obtained from Chris Schmidt, QIMR Berghofer Medical Research Institute. Cells were grown in T‐25 cell culture flasks and cultured in DMEM medium fortified with 10% fetal bovine serum (FBS) and 1% penicillin/streptomycin. Cells were primarily seeded at 5 x 10^5^ cells per T25 flask and supplemented with 5 mL media. Cells were cultured for 72 h at 37 °C with 5% CO_2_. At the end of the incubation, the growth medium was collected in 15‐mL nuclease‐free tubes. Supernatant was isolated using a dual centrifugation protocol, spinning at 300 ×  g for 20 min followed by a second spin at 4700 x g for 10 min. The samples were then stored at −80 °C until extraction. Supernatant cfDNA was extracted using QIAamp Circulating Nucleic Acid Kits (Qiagen, Hilden, Germany) and then were purified using Agentcour AMPure XP beads (Beckman Coulter, Brea, CA, USA) primarily at 0.6 : 1 bead to eluted DNA volume ratio to separate large fragment size (>700 bp). Supernatant was then transferred to another tube and further purified at 1.6 : 1 ratio to isolate fragments within the 100–300 bp range.

### 2.9. Statistics

Unpaired two‐tailed t‐test was used to compare the levels of *TERT* and *DPH3* promoter ctDNA relative to internal exonic gene region controls from cell line supernatant. Statistical analyses were performed using graphpad prism version 5.

## 3. Results

### 3.1. Mutational profile of melanoma tumors

**Figure 1:**
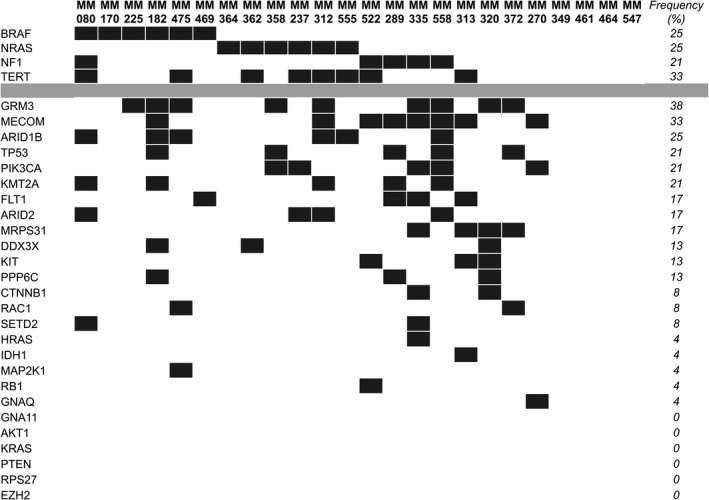
Mutational profiles of 24 FFPE melanoma tumors assessed using a custom targeted sequencing panel. The percentage of patients with alterations per gene are noted under frequency.

FFPE tumor tissue from twenty‐four stage IV melanoma was first analyzed for somatic mutations using a custom amplicon sequencing panel targeting 950 amplicons over 30 commonly mutated genes in melanoma (Table S1). Somatic mutations were identified in 20 of the samples, and four samples did not have identifiable somatic mutations within the loci analyzed (Fig. 1). Table 1 highlights the clinical characteristics of patients analyzed.

**Table 1:** Characteristics of melanoma patients with tissue and ctDNA mutational data

	*N*	Percentage
*Melanoma type*		
Cutaneous	23	96
Uveal	1	4
*Age*		
30–50	2	8
51–70	12	50
71–80	10	42
*Sex*		
Female	5	21
Male	19	79
*M classification*		
M1a	6	25
M1b	6	25
M1c	10	42
M1d	2	8
*BRAF status*		
BRAF Mutant	6	25
BRAF wild‐type	18	75
*Mutational profiling*		
Mutation found	20	83
No mutation found	4	17
*ctDNA detection at baseline*		
Positive	14	58
Negative	6	25
Not tested	4	17

Six of 24 (25%) cases had mutations in *BRAF* p.V600 (Fig. 1). The *BRAF* sequence variants identified in the tumors (*N *=* *6) via sequencing were an exact match to the variant annotated in the archival pathology reports. The uveal melanoma case (MM270) included in the study had a *GNAQ* p.Q209L mutation, which is commonly found in this melanoma subtype (Robertson *et al*., 2018). We also observed a high frequency of patients with deleterious mutations (polyPhen score >0.7) in *GRM3* (38%)*, TERT* promoter (33%), *NRAS* (25%), *NF1* (21%), and *TP53* (21%) (Fig. 1, Table S2). In patients with *TERT* promoter and *NRAS* mutations, the variants were found in the hotspot positions of the promoter region (C250T/C228T) and codon 61 (p.Q61), respectively. There were also high numbers of patients with variants in *MECOM (33%), ARID1B (25%),* and *PIK3CA (21%),* but the polyPhen scores indicated benign or tolerable effects on protein function.

Five of the 24 patients analyzed harbored cosmic‐annotated mutations in *TP53:* p.R248W, p.R248Q, p.S127F, p.S46F, and p.G266R. *GRM3* and *NF1* were mutated at 38% and 21%, respectively, with mutations distributed along the gene coding regions, consistent with their tumor suppressor nature. Four of the mutations in these genes, *GRM3* p.D548N, *GRM3* p.R668H, *NF1* p.W336T, and *NF1* p.P1851S, have been reported previously in COSMIC and/or TCGA studies of melanoma (TCGA, 2015). In line with previous reports (Cirenajwis *et al*., 2017; TCGA, 2015), patients bearing *NF1* mutations had higher median mutational burden compared to other patients in this cohort (*P *=* *0.019) (Fig. S1). The majority of mutations in these genes were not previously described, but their polyPhen scores indicate that the changes should have deleterious effects (Table S2).

Overall, our custom sequencing panel targeting commonly mutated genes in melanoma was effective in providing mutational information in most patients, allowing for identification of targetable mutations for ctDNA analyses in patients WT for *BRAF* and *NRAS*.

### 3.2. ctDNA detection in melanoma using multiple mutational targets

Once the mutational profile of tumor tissues was identified, we screened the matching plasma samples of patients for the presence of the identified mutations in ctDNA using ddPCR. The length of time between tissue biopsy and blood collection was indicated in Table S4. The selection of mutational targets for ctDNA analysis was based on the following criteria: (a) known melanoma hotspot mutation in *BRAF, NRAS,* and/or *TERT* promoter; (b) COSMIC/TCGA reported mutation; (c) other mutation with a polyPhen score >0.7 and high variant allele frequency (VAF) in the tumor.

Analysis of plasma ctDNA showed that 14 of 20 (70%) patients with mutational data had detectable ctDNA at baseline (Table 2). In cases where two mutational targets were analyzed (*N *=* *13), both mutations were found either present or absent in the ctDNA of patients, with the exception of MM362 where the *TERT* promoter mutation was not detectable in plasma. Interestingly, patients with readily detectable ctDNA were found to have multiple metastases distributed at various body sites including liver, lungs, and bones (Table 2). In contrast, patients that were ctDNA negative at baseline were found to predominantly have lymph node metastases, with the exception of MM372 which had a single lung metastasis and exclusion of patient MM270 (uveal melanoma).

**Table 2:** Levels of ctDNA at baseline and the distribution of melanoma metastasis across body sites

Patient	Mutation	Tissue	ctDNA		Mutation	Tissue	ctDNA		Sites of metastasis								
		Allelic frequency (%)	Level (c·mL^−1^)	Allelic frequency (%)		Allelic frequency (%)	Level (c·mL^−1^)	Allelic frequency (%)	*Brain*	*Bone*	*Liver*	*Lung*	*Lymph Node*	*Mesentery*	*Pancreas*	*Pelvic Region*	*Subcutaneous*
MM080	BRAF p.V600K	58	4328	38%	TERT C250T	66	1080	20		x				x			x
MM364	NRAS p.Q61K	63	2064	34%							x	x	x	x		x	
MM225	BRAF p.V600E	12	1695	38%						x		x				x	
MM475	BRAF p.V600R	46	380	9%	RAC1 p.P29S	48	446	12		x	x	x	x				
MM469	BRAF p.V600E	24	233	8%							x						
MM182	BRAF p.V600K	43	540	15%	GRM3 p.P605S	54	720	12						x	x	x	
MM362	NRAS p.Q61K	89	390	7%	TERT C250T	48	0	0		x		x	x	x			x
MM555	NRAS p.Q61L	46	98	4%	TERT C250T	24	16	2	x	x		x	x				
MM522	KIT p.L576P	29	56	4%	TERT C228T	23	11	4			x	x	x				
MM289	TP53 p.R248Q	36	48	1%	FLT1 p.T543I	36	18	1			x						
MM170	BRAF p.V600E	26	33	1%									x	x			x
MM358	NRAS p.Q61K	61	22	5%	TP53 p.S127F	80	20	3		x		x	x				x
MM320	KIT p.V599A	49	13	1.2%	DDX3X p.R475C	23	2	0.1		x				x			
MM237	NRAS p.Q61K	26	4	0.1%	PIK3CA p.S326F	36	9	1				x					
MM313	KIT p.W557R	69	0	0	TERT C228T	44	0	0					x				x
MM372	RAC1 p.P29S	59	0	0								x					
MM312	NRAS p.Q61R	50	0	0	TERT C250T	37	0	0					x				
MM335	CTNNB1 p.D32N	12	0	0	NF1 p.P1851S	22	0	0					x				
MM558	TP53 p.R248W	24	0	0									x				
MM270	GNAQ p.Q209L	28	0	0						x		x					x

### 3.3. Concordance of mutations in tissue and plasma

Concordance of mutations in tissue and plasma was then further analyzed in 7 of the patients who had detectable ctDNA and sufficient plasma available (Table 2). The mutation profile of these plasma samples was determined using a custom sequencing panel, targeting the same loci as the panel used to analyze FFPE tumor tissues but incorporating molecular unique identifiers to enable the detection of low frequency mutations in plasma ctDNA (Table S3).

On average, 89% (range 75–100%) of SNVs found in the plasma of patients by targeted sequencing were also detected in the tumor tissue (Fig. S2). Only three mutations found in plasma were not identified in the matching tumor (Table 3). If mutations that were in the tissue but not in plasma are included in the concordance evaluation, that is, the number of overlapping SNVs relative to the overall number of plasma *plus* tissue mutations, average concordance is reduced to 67%, with a range of 30–100% (Table 3 and Fig. S2). In particular, *TERT* promoter mutations were not detected in plasma by NGS, but detected by ddPCR in four of the five discordant cases (Table 2). The *TERT* promoter region is difficult to amplify due to its high GC content, and the mutant reads from this locus were below threshold in the NGS analysis. If positivity by ddPCR is included in the concordance analysis between mutations found in plasma and tissues, the overall concordance between plasma and tissue biopsies is 80% (range 40–100%).

**Table 3:** Single nucleotide variation profile derived from NGS of 7 melanoma patients with matched FFPE tissue and plasma

	MM080		MM358		MM362		MM469		MM475		MM522		MM555	
	ctDNA	Tissue	ctDNA	Tissue	ctDNA	Tissue	ctDNA	Tissue	ctDNA	Tissue	ctDNA	Tissue	ctDNA	Tissue
ARID1B p.P1491L		4												
ARID1B p.P1006L									2	17				
ARID1B p.I1666T													7	48
ARID2 p.T969I		4												
BRAF p.V600K	25	58												
BRAF p.V600E							8	30						
BRAF p.V600R									8	46				
DDX3X p.I195N					3	85								
FLT1 p.G706E							3	4						
FLT1 p.T335P							6	40						
GRM3 p.D548N			4	61										
GRM3 p.D744N									1	14				
KIT p.L576P											2	29		
KMT2A p.R1630Q	1													
KMT2A p.D2893E		5												
MAP2K1 p.P124S									4	19				
MECOM p.S419F	15	15												
MECOM p.R748Q											2	25		
MECOM p.P701S											1	24		
NF1 p.I1624L	9	11												
NF1 p.P1421L		3												
NF1 p.L792F							3				2			
NRAS p.Q61K			5	61	7	89								
NRAS p.Q61L													5	46
RAC1 p.P29S									8	48				
SETD2 p.S917N		49												
TERT C228T									2^a^	60	4^a^	23		
TERT C250T	20^a^	66				48							2^a^	24
TP53 p.S127F			1	80										
Concordance (NGS only)	30		100		67		75		83		60		67	
Concordance (NGS + ddPCR)	40		100		67		75		100		80		100	

Boxes denote frequency abundance (%) of each SNV detected by NGS in tissue only (blue), plasma only (light red), or in both biopsies (dark red).

a Denotes SNV in ctDNA detected by ddPCR but not NGS.

The major contributor to discordance was case MM080, with 5 SNVs found in tumor but not in plasma. In this case, there was a 3‐year gap between tissue and blood sampling, and thus, the high number of mutations found only in the tumor is possibly the result of clonal evolution. Overall, these data indicate that ctDNA is readily detectable in stage IV melanoma patients with multiple metastatic sites. The high detection rate of tumor‐associated mutations in plasma prior to treatment further reinforces the utility of ctDNA for genetic profiling as a potential surrogate for solid tumor biopsy.

### 3.4. ctDNA monitoring of melanoma patients using single or multiple mutational targets

**Figure 2:**
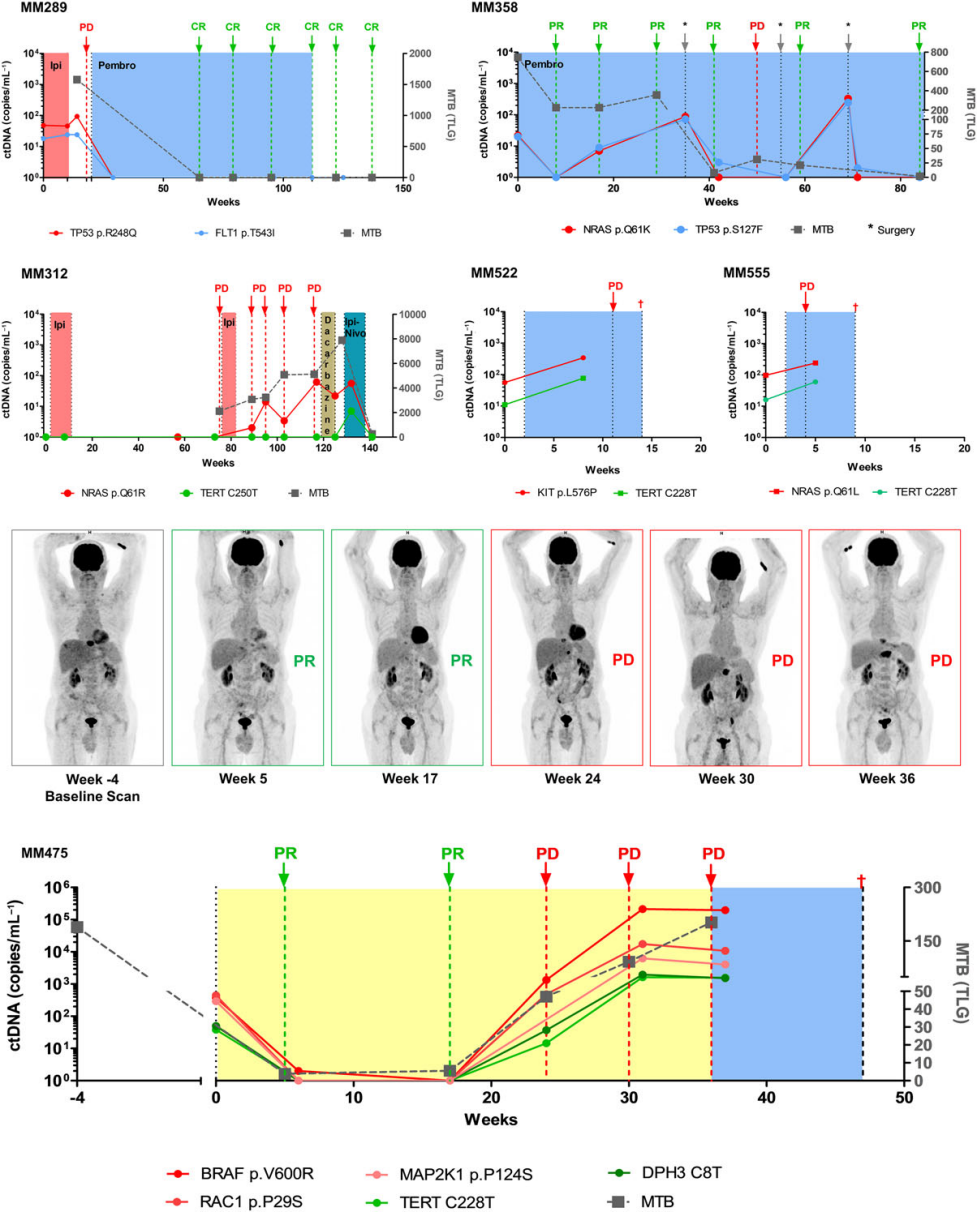
Monitoring ctDNA levels in melanoma patients undergoing systemic therapy. Plasma ctDNA levels were determined using two mutations and compared to FDG‐PET metabolic tumor burden (MTB). Therapies are indicated by colored boxes. Disease status by radiological imaging is indicated by arrows and labeled as PR: partial response, PD: progressive disease, or CR: complete response. For patient MM475, PET scan images corresponding to clinical responses are shown above.

To investigate the utility of ctDNA as a surveillance biomarker in melanoma patients undergoing systemic therapy, patients were monitored longitudinally for ctDNA via ddPCR targeting multiple mutations using the selection criteria described above. In the case of MM312, who presented with isolated nodal disease in the groin, *NRAS* p.Q61R mutation was undetectable in plasma ctDNA at baseline but became detectable upon further progression of disease (PD) (Fig. 2). Nonetheless, ctDNA was only detectable when MTB had almost doubled at week 90, suggesting a potential effect of MTB on ctDNA detection. No decrease in ctDNA was observed in this patient at week 132 after commencing ipilimumab/nivolumab therapy, but a significant decline in ctDNA was observed at week 141. Of note, the *TERT* promoter mutation was undetectable in all but one of the plasma collections.

When two or more mutations were tracked during treatment, the ctDNA kinetics of these mutations showed overlapping or parallel curves that were similarly consistent with clinical response (Fig. 2), irrespective of whether they were bona fide melanoma drivers (*BRAF*,* NRAS,* and/or *TERT*) or rare deleterious mutations in melanoma (*TP53 p.R248Q, FLT1 p.T543I, KIT p.L576P*). In general, changes in ctDNA levels corresponded with the changes in MTB of patients during treatment (Fig. 2). For example, patient MM475 (Fig. 2) had multiple recognized melanoma driver mutations including *BRAF* p.V600R, *RAC1* p.P29S, *MAP2K1* p.P124S, *TERT* C228T, and *DPH3* C8T. All of these mutations decreased in concordance with response to *BRAF* and *MAPK* inhibition and correlated with a declining MTB. The concentration of all five mutations greatly increased in plasma at week 23, corresponding to a small increase in MTB. At progression (week 24), *BRAF* p. V600R was at 10‐fold higher concentration than *RAC1* and *MAPK2* mutations, suggesting a gain in copy number, which is a common mechanism of resistance to *BRAF* inhibition (Johnson *et al*., 2015).

These results indicate that well‐known driver mutations or infrequent deleterious mutations can be used for ctDNA‐based patient surveillance, given their close correlation with changes in MTB.

### 3.5. TERT ctDNA is represented in lower levels in plasma

**Figure 3:**
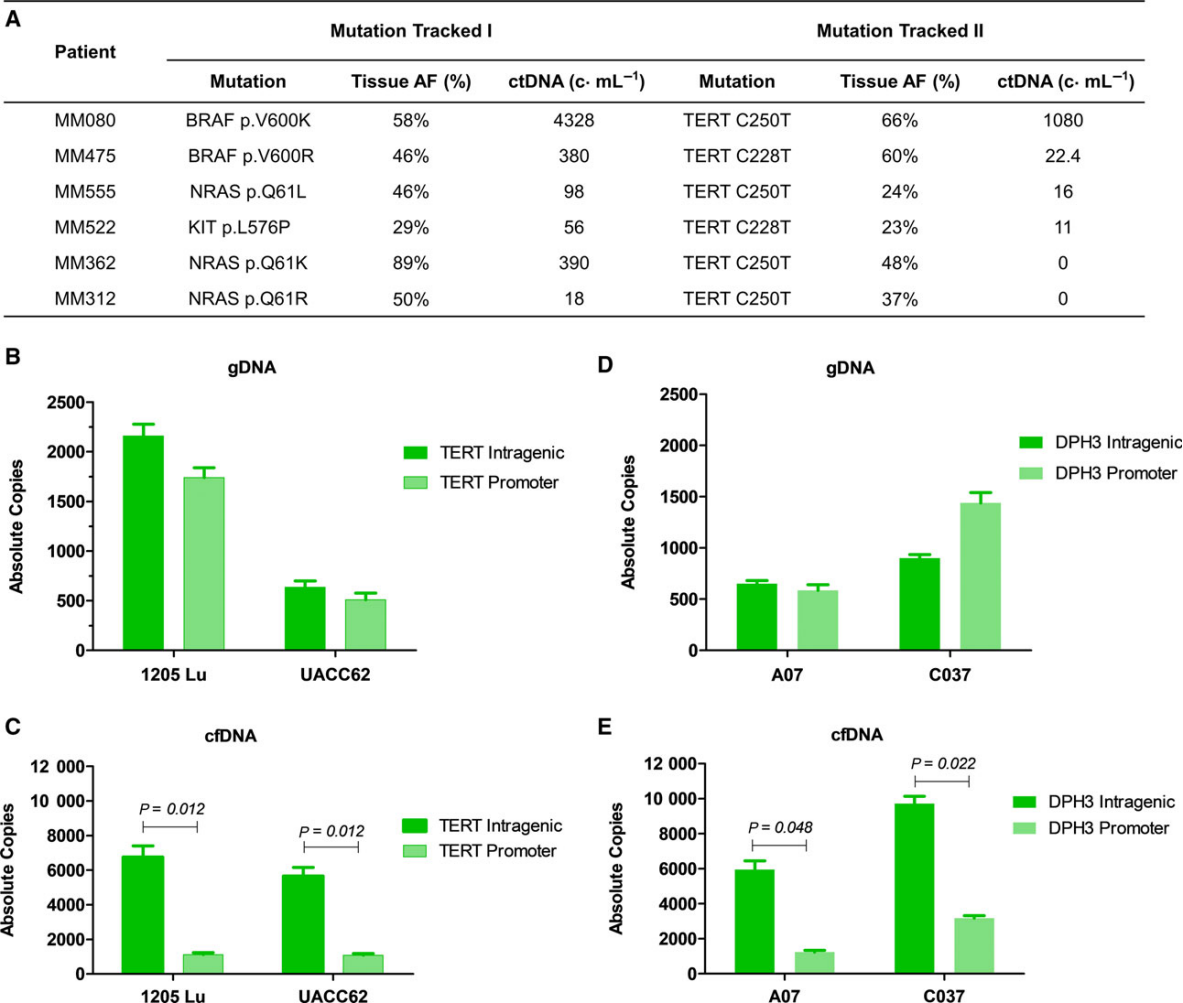
Differential levels of promoter mutations in ctDNA. (A) Comparison of the *TERT* promoter mutations allelic frequency (AF) in tumor tissue and copies per mL of plasma relative to the major driver mutation in six melanoma patients. (B–E) Bar graphs of the absolute copy number of intragenic or promoter region of *TERT* in 1205Lu and UACC62 gDNA and supernatant ctDNA (B, C) or intragenic or promoter region of *DPH3* in C032 and A07 gDNA or supernatant ctDNA (D, E). Standard deviations of triplicate experiments are indicated. *P* values ≥0.05 (unpaired *t*‐test) were considered as statistically significant.

Longitudinal monitoring of patients with detectable *TERT* ctDNA (Fig. 2) revealed lower copies of these mutations in plasma compared to that of other driver mutations. In fact, of the 6 patients confirmed to harbor *TERT* promoter mutations as well as another driver mutation in their tumor tissue, *TERT*‐mutated ctDNA was underrepresented or undetectable in plasma relative to the levels of the other mutation analyzed (Fig. 3A). Given the location of these mutations within a promoter region, we hypothesized that the underrepresentation of *TERT* copies in plasma ctDNA is a result of low nucleosome occupancy at these sites, providing a lack of protection of this region against nuclease cleavage during cell apoptosis (Ulz *et al*., 2016).

We therefore conducted *in vitro* experiments using the melanoma cell lines 1205Lu and UACC62, which are known to carry *TERT* promoter C228T and C250T mutations, respectively, to determine whether similar patterns of underrepresentation of the *TERT* promoter regions are observed in the DNA isolated from the supernatant of these cell lines. We found no difference in the ratio of absolute copies of the intragenic *TERT* and the promoter region in the genomic DNA of these cells (Fig. 3B). However, there were significant differences in the copies of promoter *TERT* vs intragenic *TERT* in supernatant‐derived ctDNA for both cell lines (*P = 0.012*), with more than fivefold less *TERT* promoter copies compared intragenic *TERT* (Fig. 3C).

Recently, mutations in the *DPH3* promoter region have been found in 10% of melanomas (Denisova *et al*., 2015). Given its location, we determined whether the *DPH3* promoter region is also underrepresented in plasma. We screened our patient cohort for *DPH3* mutations, and similarly, we found underrepresentation of this promoter mutation in the blood of patient MM475 (Fig. 2). To further validate these findings *in vitro*, we then tested the supernatant from A07 and C032 melanoma cell lines that were found to carry the *DPH3* C8T promoter mutation. We again found a significantly lower number of absolute copies of *DPH3* promoter compared to exonic *DPH3* in the supernatant cfDNA of A07 (*P = 0.048)* and C032 (*P = 0.022)* cell lines (Fig. 3D). The overall copies of exonic and promoter regions of *DPH3* were found at approximately similar levels in genomic DNA from the cells (Fig. 3E). Notably, these experiments highlight the variability in representation of different loci in cfDNA, which can impact the detection of promoter region mutations in plasma ctDNA.

## 4. Discussion

Multiple studies over the last three years have provided increasing evidence of the value of ctDNA for monitoring treatment response in metastatic melanoma patients (Ascierto *et al*., 2013; Girotti *et al*., 2016; Gray *et al*., 2015; Knol *et al*., 2016; Schreuer *et al*., 2016) but mostly relied on a few common driver mutations to determine disease status for ctDNA analysis. Therefore, improved methods that allow interrogation of multiple genes together with further studies are required to determine the concordance of genetic aberrations in matched tissue and plasma biopsies in melanoma patients.

In this study, we validated a targeted sequencing panel, comprised of 30 melanoma‐associated genes, and compared the mutation profiles in tumor tissue and plasma across multiple patients prior to therapy commencement. We demonstrated a high level of concordance between tissue and plasma biopsies, supporting the use of ctDNA as a suitable surrogate for genetic profiling. We reported on the kinetics of ctDNA using multiple targeted mutations within the same patient throughout treatment response and disease progression. Importantly, we provided clinical and *in vitro* evidence of the underrepresentation in ctDNA of mutations in promoter regions such as those of *TERT* and *DPH3*. All these findings need to be considered for the clinical implementation of ctDNA as a monitoring tool for melanoma. Our results are of particular significance for patients who are negative for *BRAF* mutations, as we show that other mutations can be used for tumor monitoring.

Given the high mutational heterogeneity of melanoma tumors, the use of comprehensive and targeted NGS technologies for molecular profiling proved highly beneficial. With these, we characterized the landscape of mutations in the tissue and peripheral blood to identify molecular targets for patient surveillance. Our panel was able to identify clinically relevant somatic variants in 83% of patients, suggesting high efficiency for identifying targetable mutations for longitudinal ctDNA monitoring.

We found a high proportion of metastatic melanoma patients had detectable ctDNA at baseline and that genomic alterations in peripheral blood in ctDNA‐positive patients were highly concordant with those in the tissue. We acknowledge that our conclusions are based on a small sample size, including only seven tissue and plasma ctDNA pairs for NGS analysis. However, our results add to the mounting evidence on the potential utility of ctDNA as a surrogate to solid tumor biopsy and as an ideal candidate for molecular analysis as previously demonstrated for various cancers (Chae *et al*., 2017; Goldberg *et al*., 2018; Jovelet *et al*., 2016; Murtaza *et al*., 2015; Wong *et al*., 2017). Notably, we observed a bias towards ctDNA detectability in patients with high metastatic burden. Patients with an isolated metastasis, particularly in the lymph nodes, consistently had no detectable ctDNA at baseline, which constitutes a limitation to the use of plasma ctDNA in the clinic (De Mattos‐Arruda *et al*., 2015; Li *et al*., 2016; Momtaz *et al*., 2016).

It is also important to note that we found few SNVs in plasma only, which is in line with the idea that ctDNA is representative of the sum of the multiple tumor lesions and clones. While we did not assess the mutational profile of multiple metastases, this result is consistent with studies in melanoma and other cancers detailing the capacity of ctDNA to comprehensively capture tumor heterogeneity (Bettegowda *et al*., 2014; FitzGerald *et al*., 2017; Murtaza *et al*., 2015; Wong *et al*., 2017). Our results also highlighted high discordance in the mutational profile of a patient with a large gap between tissue and blood collection, suggesting potential impact of clonal evolution on the concordance of tissue and plasma biopsies. Thus, changes in the somatic mutational landscape, as part of disease evolution, must be taken in consideration when monitoring cases where new metastases are inaccessible and selection of targetable mutations for monitoring melanoma patients or treatment selection depends on the primary tumor.

Notably, the kinetics of multiple ctDNA targets, particularly melanoma driver mutations, uniformly informed on tumor dynamics in response to treatment. These results underscore the fact that well‐known and/or rare driver mutations can be used for ctDNA quantification and patient surveillance. These findings are in contrast to a previous study by Gremmel *et al*. describing a case of mucosal melanoma with two distinct tumor subclones, identified by whole exome sequencing, with differential response to imatinib, chemotherapy, and immunotherapy (Gremel *et al*., 2016). However, the use of a limited number of targeted loci in our study may have constrained our ability to fully capture tumor heterogeneity. Another limitation of our study is that only a single metastasis was analyzed from each patient to obtain the mutational data used for ctDNA surveillance. While the ctDNA kinetics of driver mutations may be used to determine systemic response to treatment, there is a possibility that subclones prevalent in other metastases may serve as a good indicator of the specific response of individual metastatic deposits to therapy.

Significantly, our data call for caution when interpreting ctDNA levels based on single locus analysis, particularly in the context of promoter mutation targets. Previous research has observed the presence of lower copies of *TERT* ctDNA relative to other mutations (McEvoy *et al*., 2017; Wong *et al*., 2017). Herein, we also found a similar pattern of *TERT* and *DPH3* promoter underrepresentation in cell‐free DNA *in vivo* and *in vitro*. Ulz *et al*. (2016) previously reported that promoters of transcriptionally active genes are often devoid of nucleosomes and can induce variability in plasma cfDNA. Thus, the biological process of cfDNA biogenesis may significantly affect quantitative‐based applications for liquid biopsy, particularly for patient monitoring. Nevertheless, as mutations in the *TERT* promoter enhance *TERT* expression, which is associated with poor disease‐free and melanoma‐specific survival (Nagore *et al*., 2016), low levels of *TERT* promoter mutation in cfDNA relative to other activating mutations may be harnessed to delineate patients with transcriptionally active *TERT*. Overall, our results underscore the need for further studies into ctDNA biology prior to its clinical implementation.

## 5. Conclusions

Overall, ctDNA has significant clinical value as a noninvasive source of genetic material for mutational analyses, which can guide treatment selection, and for identification of traceable markers for patient monitoring in melanoma. Its ease of access and relative ability to accurately reflect disease burden make it a particularly reliable biomarker for the surveillance of melanoma patients during treatment course.

## Conflict of interest

The authors declare no conflict of interest.

## Author contribution

LC, EG, and MZ conceived and designed the project. LC, EG, GT, CR, and BA conducted the experiments and analysis of the results. AR and MR were involved in sample preparation and analysis of results. NH provided the DPH3‐mutant cell lines and contributed to the study design. MM, MAK, and TMM recruited the patients. MM, MAK, TMM, LW, ZA, and AM helped in clinical data analysis and provided feedback on patient clinical status. All authors provided feedback, revised, and approved the final manuscript.

## Supporting information


**Fig. S1.** Comparison of number SNVs in patients with and without NF1 mutations.
**Fig. S2.** Percent concordance of SNVs in tissue and plasma biopsies by NGS or NGS plus ddPCR.

**Table S1.** Melanoma panel.

**Table S2.** NGS mutational profile of study cohort.

**Table S3.** Comparison of SNVs in tissue and plasma biopsies.

**Table S4.** Time‐lapse of tissue and plasma biopsies (weeks) obtained for the study cohort.

**Table S5.** Specificity of ddPCR assays.
