# Supplementary material for: Locus‐specific concordance of genomic alterations between tissue and plasma circulating tumor DNA in metastatic melanoma
Source: Mol Oncol. 2018 Dec 7;13(2):171–84. doi: 10.1002/1878-0261.12391 (PMC6360370; doi:10.1002/1878-0261.12391)
Supplement: Supplementary file 1 — Fig. S1. Comparison of number SNVs in patients with and without NF1 mutations. Fig. S2. Percent concordance of SNVs in tissue and plasma biopsies by NGS or NGS plus ddPCR. [file MOL2-13-171-s001.DOCX]

**Figure S1: Comparison of number SNVs in patients with and without NF1 mutations.** Graphs indicate a statistically significant difference (*P*=0.0219) in mutational burden between tumours with and without NF1 mutations.

**Figure S2: Percent concordance of SNVs in tissue and plasma biopsies by NGS or NGS *plus* ddPCR.** Each point represents the percent concordance within a patient. The mean and standard deviation are indicated for each set.
